# Identification of adolescent girls and young women for targeted HIV prevention: a new risk scoring tool in KwaZulu Natal, South Africa

**DOI:** 10.1038/s41598-020-69842-x

**Published:** 2020-08-03

**Authors:** Sarah Gabrielle Ayton, Martina Pavlicova, Quarraisha Abdool Karim

**Affiliations:** 1 Mailman School of Public Health, Columbia University, 722 W. 168th Street, 6th floor, Rm. 635, New York, NY 10032 USA; 2 Escuela de Medicina y Ciencias de la Salud, Tecnologico de Monterrey, Monterrey, Mexico; 3 Centre for the AIDS Programme of Research in South Africa (CAPRISA), University of KwaZulu-Natal, Durban, South Africa

**Keywords:** Statistical methods, Risk factors, HIV infections, Prognostic markers, Epidemiology

## Abstract

The ongoing spread of human immunodeficiency virus (HIV) has driven novel interventions, such as antiretrovirals, for pre-exposure prophylaxis. Interventions have overlooked a high-risk Sub-Saharan African population: adolescent girls and young women (AGYW), particularly those under 18. We apply the Balkus risk tool among rural South African AGYW (n = 971) in a hyper-endemic setting, identify limitations, and assess deficiencies with modern statistical techniques. We apply the “Ayton” tool, the first risk tool applicable to sub-Saharan African AGYW, and compare performance of Balkus and Ayton tools under varying conditions. The Ayton tool more effectively predicted HIV acquisition. In low and high-risk AGYW, the Ayton tool out-performed the Balkus tool, which did not distinguish between risk classes. The Ayton tool better captured HIV acquisition risk and risk heterogeneities due to its AGYW-focused design. Findings support use of the Ayton tool for AGYW and underscore the need for diverse prognostic tools considering epidemic severity, age, sex and transmission.

*Clinical Trial Number* ClinicalTrials.gov (NCT01187979) and the South African National Clinical Trials Registry (SANCTR) (DOH-27-0812-3345).

## Introduction

Prognostic tools aid the identification of high-risk candidates for preventative interventions by calculating risk of health events for a given patient^1–3^. While risk calculators have traditionally targeted chronic disease, advancements in statistical modeling and ongoing outbreaks have warranted the development of risk prediction tools for infectious diseases^2^. Human immunodeficiency virus (HIV) and acquired immunodeficiency syndrome (AIDS) risk calculators prioritize populations at the highest risk of infection, namely sub-Saharan African women and men who have sex with men (MSM)^4–6^, as well as injecting drug users^6,7^. Identifying individuals at high-risk of HIV could enable health care providers to efficiently prioritize provision of pre-exposure prophylaxis (PrEP), a medical regimen that prevents HIV infection^8–13^.

In high-prevalence hyper-endemic settings, such as eastern and southern Africa, adolescent girls and young women (AGYW), aged 14–25 years, are particularly vulnerable to HIV acquisition due to power imbalance in sexual relationships^14–19^, particularly those in which HIV is acquired from men over 25 years old^15,18–21^, and biological factors (i.e., increased genital inflammation)^18^. Despite their elevated risk of infection, AGYW, particularly those under 18 years, underutilize health services for fear of stigmatization^18,19^. Due to the disproportionate burden of HIV/AIDS among African AGYW, efficient identification of females with high HIV risk and an understanding of AGYW risk heterogeneities is critical for effective targeting of prevention interventions^7,15,20–26^. Specific HIV risk tools have been developed for adult African women^4,5^ and one tool has been developed for South African AGYW^27^. However, the widely used tools developed for women exclude AGYW under 18 years, leaving the population vulnerable and underserved.

In this manuscript, we focus on the Balkus HIV risk prediction tool^4^ and the Ayton AGYW risk prediction tool^27^. While preliminary validation of the Balkus tool has indicated high predictive sensitivity among African women over 18 years of age^4^, it is unknown whether it will perform as well in AGYW in the same setting. AGYW may acquire HIV prior to adulthood, and AGYW-specific risk behaviors influence adult HIV risk^7^. The exclusion of AGYW from validation of risk prediction tools, such as the Balkus tool, overlooks their role in HIV transmission and ignores their particular transmission vulnerabilities. First, we applied the Balkus tool to a sample of South African AGYW and evaluated the tool’s ability to predict AGYW HIV serostatus at 1 year using raw scores and imputed scores via advanced statistical simulation techniques. Second, the Ayton AGYW tool classified risk in the sample and its ability to predict HIV serostatus at 1 year was also evaluated. Risk predictive power was compared between the Balkus tool and the Ayton tool, and we assessed the Balkus tool’s ability to distinguish between Ayton risk classes.

## Results

The CAPRISA 007 (CAP007) study captured 1069 AGYW participants who were followed up at 1 year (Table 1). Among these participants, 20 were HIV seropositive and 111 were herpes simplex virus (HSV-2) seropositive at baseline (2011); by 2012, there were 18 additional AGYW who became HIV seropositive. Most HIV seronegative AGYW participants did not report use of contraception, drugs, or alcohol in the past year and did not report past-year pregnancy. AGYW participants in our sample demonstrated high knowledge about HIV, had been previously tested for HIV, and only missed school due to illness; these participants further self-identified as being at lower risk of infection and were under 18 years old.

**Table 1:** Demographic characteristics of AGYW CAP007 participants (n = 1069; 2011–2012).

Characteristics	Overall (n = 1069)	HIV + (n = 20)	HIV− (n = 1049)
	Frequency (%)	Frequency (%)	Frequency (%)
**Age (years)**			
Median (IQR)	17.00 (16.00–18.00)	17.50 (17.00–18.25)	17.00 (16.00–18.00)
Mean (95% CI)	16.92 (16.82, 17.02)	17.85 (17.03–18.67)	16.90 (16.80–17.01)
**Age**			
$\ge$ 18	302 (28.25)	10 (50.00)	292 (27.84)
< 18	767 (71.75)	10 (50.00)	757 (72.16)
**Financial dependence**			
Yes	117 (10.94)	3 (15.00)	114 (10.87)
No	952 (89.06)	17 (85.00)	935 (89.13)
**Drug use (past year)**			
At least once	39 (3.65)	1 (5.00)	38 (3.62)
Never	1016 (95.04)	19 (95.00)	997 (95.04)
**Alcohol use (past year)**			
At least once	164 (15.49)	4 (20.00)	160 (15.25)
Never	895 (84.51)	16 (80.00)	879 (83.79)
**Pregnancy**			
Yes	44 (4.14)	1 (5.00)	43 (4.10)
No	1019 (95.86)	19 (95.00)	1000 (95.33)
**School absence (past year)**			
Illness	326 (30.50)	7 (35.00)	319 (30.41)
Illness and other reason	216 (20.21)	6 (30.00)	210 (20.02)
Other reason	181 (16.93)	4 (20.00)	177 (16.87)
None	346 (32.37)	3 (15.00)	343 (32.70)
**HIV knowledge**			
Low	91 (8.51)	–	91 (8.67)
High	978 (91.49)	20 (100.00)	958 (91.33)
**Prior HIV testing**			
Yes	934 (88.11)	15 (75.00)	919 (87.61)
No	126 (11.89)	5 (25.00)	121 (11.53)
**Contraception (past year)**			
Condom use	277 (25.91)	7 (35.00)	270 (25.74)
Other contraception	404 (37.79)	3 (15.00)	204 (19.45)
None	585 (54.72)	10 (50.00)	575 (54.81)
**Perceived HIV risk**			
High risk	80 (7.48)	2 (10.00)	78 (7.44)
Low risk	404 (37.79)	5 (25.00)	399 (38.04)
Not at risk	585 (54.72)	13 (65.00)	572 (54.53)
**HSV-2 status**			
Positive	94 (8.79)	4 (20.00)	90 (8.58)
Negative	902 (84.38)	12 (60.00)	890 (84.84)
**HIV status (at one year)**			
Positive	38 (3.55)	20 (100.00)	18 (1.72)
Negative	1031 (96.45)	–	1031 (98.28)

Raw Balkus scores were computed using age, HSV-2, alcohol use, and financial support variables. The CAP007 study did not capture three risk predictors among AGYW, which are not applicable to the population and therefore were not measurable: ${X}_{2}$ (marital and cohabitation status), ${X}_{5}$ (primary sex partners with other partners), and ${X}_{6}$ (curable STI). These variables were excluded in calculation of raw Balkus scores, and were simulated to enable full Balkus score calculation using generic and reality-based simulations of variable prevalence. 971 AGYW had complete observations across all items evaluated in the raw Balkus tool.

### Balkus tool: evaluation of raw scores

**Figure 1:**
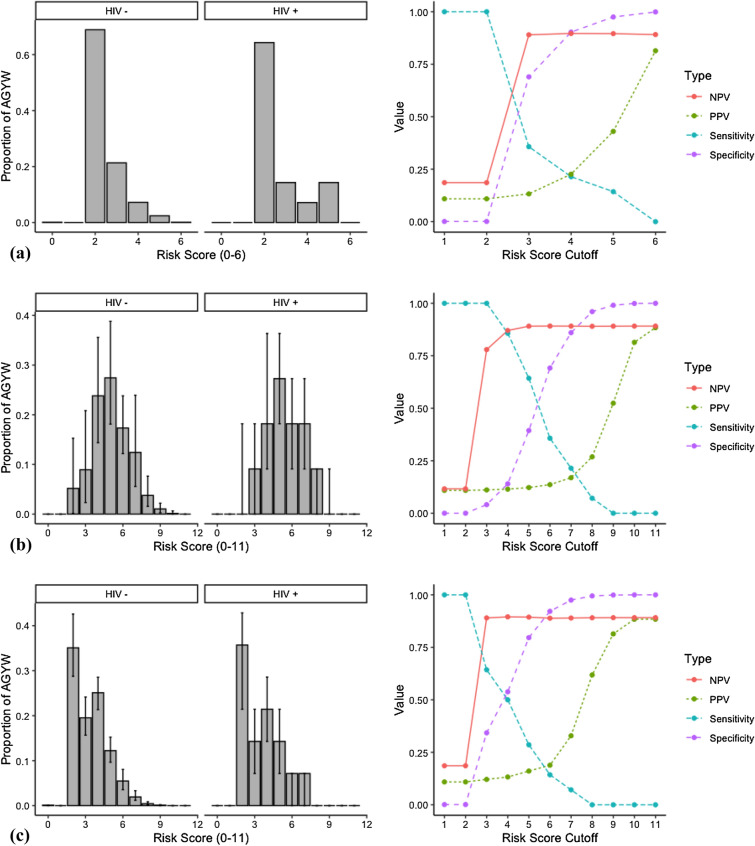
Evaluation of raw and simulated Balkus scores in AGYW. Distribution of (**a**) raw, (**b**) generic simulated (median [IQR]), and (**c**) reality-based simulated (median [IQR]) Balkus risk scores in AGYW who remained HIV seronegative and those who became seropositive at 1 year (left, Supplementary Table S1). Balkus scores were evaluated for sensitivity, specificity, PPV, and NPV against 1-year HIV status (right, Supplementary Table S2).

In preliminary analyses, observed variables were used to compute the raw Balkus scores for our sample of AGYW indicating a high frequency of low scores (Supplementary Table S1, Fig. 1). Most HIV seroconversions (n = 9) were observed to have a score of 2 (HIV prevalence: 0.01, 95% CI: 0.01–0.03), although HIV prevalence was highest among those with a score of 5 (n = 2, HIV prevalence: 0.08, 95% CI: 0.01–0.28). As score cutoff value increased, sensitivity decreased while specificity, positive predictive value (PPV), and negative predictive value (NPV) increased (Supplementary Table S2, Fig. 1). Sensitivity (1.00, 95%CI: 0.77–1.00) was highest at the cutoff values of 1 and 2, while specificity (1.00, 95%CI: 0.99–1.00), PPV (0.81, 95%CI: 0.45–0.96), and NPV (0.89, 95%CI: 0.88–0.91) were highest at the cutoff value of 6. Based on all four criteria, a value of 3 is the best cutoff in our raw Balkus score assessment.

Results from the development and validation of the Balkus tool indicated higher sensitivity and NPV and lower specificity and PPV in the VOICE cohort (sensitivity: 0.98, specificity: 0.15, PPV: 0.06, NPV: 0.99) and in the HPTN 035 cohort (sensitivity: 0.84, specificity: 0.46, PPV: 0.05, NPV: 0.99), where results were presented for a cutoff value of 3, compared with the raw Balkus score evaluation for the same cutoff value of 3. Evaluation at the higher cutoff value of 5 reported for the VOICE cohort (sensitivity: 0.91, specificity: 0.38, PPV: 0.08, NPV: 0.99) and for the HPTN 035 cohort (sensitivity: 0.58, specificity: 0.71, PPV: 0.07, NPV: 0.98), indicated higher sensitivity and NPV, and lower specificity and PPV, than those observed in the raw Balkus score evaluation for the same cutoff value of 5.

### Balkus tool: evaluations of generic and reality-based simulated scores

Evaluation of the Balkus tool, using 100,000 generic scenario simulations (prevalence 0.0–1.0) and 100,000 reality-based scenario simulations (prevalence 0.0–0.4) of excluded variables, resulted in a wider distribution of scores, ranging from 0 to 11 (Supplementary Table S1, Fig. 1). In generic simulations, most HIV seroconversions had a score between 4 and 7, while highest HIV prevalence was observed in those with a score of 10 (0.08, 95%CI: 0.00–0.72). In reality-based simulations, most HIV seroconversions were observed with scores between 2 and 5, while highest HIV prevalence was observed in those with a score of 10 (0.07, 95%CI: 0.00–0.99).

In both generic and reality-based simulations, sensitivity increased with score cutoff, while specificity, PPV, and NPV decreased (Supplementary Table S2, Fig. 1). Sensitivity was highest at cutoff values of 1 and 2 in generic (1.00, 95%CI: 0.77–1.00) and reality-based simulations (1.00, 95%CI: 0.77–1.00). In both simulations, specificity (generic: 1.00, 95%CI: 1.00–1.00; reality-based: 1.00, 95%CI: 1.00–1.00) and PPV (generic: 0.88, 95%CI: 0.50–0.98; reality-based: 0.88, 95%CI: 0.50–0.98) were highest at the cutoff value of 11. NPV was highest at the cutoff value of 6 (0.89, 95%CI: 0.83–0.92) in the generic scenario simulation, and highest at the cutoff value of 4 (0.90, 95%CI: 0.83–0.94) in the reality-based scenario simulation, after which the NPV remained constant. Based on sensitivity, specificity, PPV, and NPV, a value of 6 is the best cutoff in our Balkus score under the generic scenario simulation and a value of 4 is the best cutoff in our Balkus score under the reality-based scenario simulation.

Compared with the reported results from the VOICE cohort with a cutoff value of 3, generic scenario simulations and reality-based simulations yielded lower sensitivity, specificity, and NPV. The same was true of the reality-based simulations compared with the HPTN 035 cohort with a cutoff value of 3, while generic simulations had higher sensitivity as well as PPV. Generic simulations resulted in a higher mean sensitivity compared with the HPTN 035 cohort and lower mean sensitivity than reported for both the VOICE cohort, with a cutoff of 5; mean specificity in generic simulations was lower than that observed in both VOICE and HPTN 035 cohorts. Reality-based simulations resulted in a higher mean specificity and lower mean sensitivity than that reported in both HPTN 035 and VOICE cohorts, with a cutoff value of 5. Both generic and reality-based scenario simulations resulted in higher mean PPVs and lower NPVs compared with both VOICE and HPTN 035 cohorts, with the score cutoff value of 5.

### Ayton tool evaluation

Evaluation of the Ayton tool AGYW risk classes with HIV status at 1 year resulted in highest sensitivity (0.60, 95%CI: 0.32–0.84) and moderate specificity (0.58, 95%CI: 0.55–0.61) when comparing the low risk classification to almost no risk classification (reference group) (Table 2). The highest specificity (0.84, 95%CI: 0.81–0.87) and moderate sensitivity (0.33, 95%CI: 0.07–0.70) was observed when comparing the high-risk classification to the almost no risk group.

**Table 2:** Evaluation results of Ayton tool in predicting HIV serostatus at 1 year in AGYW (n = 971; 2011–2012).

Risk class	Sensitivity	Specificity	PPV*	NPV*
Almost no risk (Ref.)	–	–	–	–
Low risk	0.60 (0.32, 0.84)	0.58 (0.55, 0.61)	0.16 (0.11, 0.22)	0.92 (0.86, 0.95)
High risk	0.33 (0.07, 0.70)	0.84 (0.81, 0.87)	0.22 (0.10, 0.41)	0.91 (0.86, 0.94)

*Computed with 11.4% prevalence estimate of HIV in South African AGYW (Mabaso et al., 2018).

### Comparisons of Balkus tool scores (raw and simulated) versus Ayton tool scores

**Figure 2:**
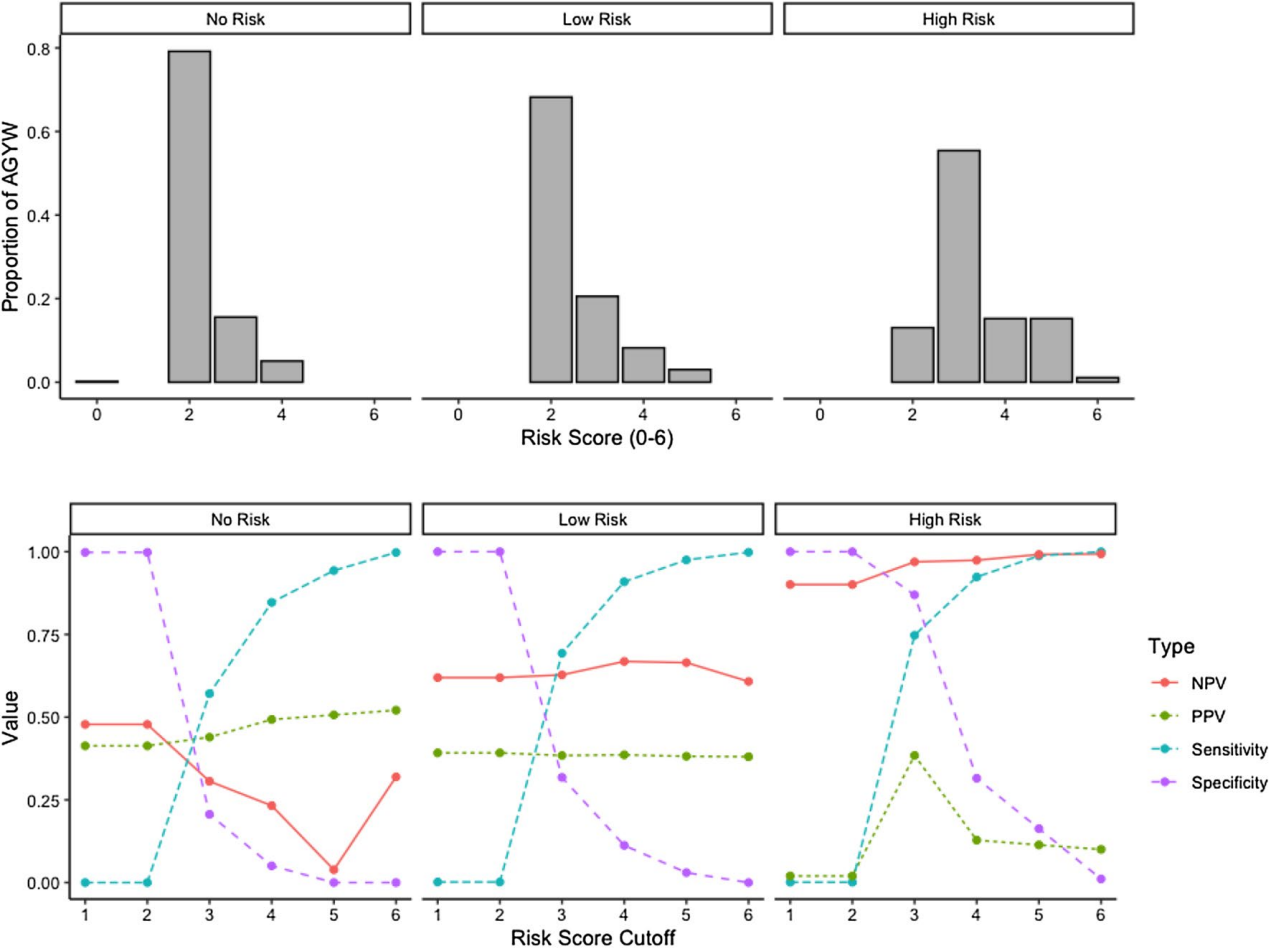
Comparison of risk classes from the Ayton with raw Balkus scores. Distribution of raw Balkus risk scores in AGYW who were classified by the Ayton tool as almost no, low, and high risk of HIV acquisition at 1 year (above). Raw Balkus scores were evaluated for sensitivity, specificity, positive predictive value, and negative predictive value against risk class determined by the Ayton tool (below).

Raw Balkus scores were computed for each Ayton AGYW tool risk class and tested against risk classification, revealing that most AGYW, across all risk classes, scored values of 2 or 3 (Fig. 2). Computed sensitivity and specificity for the Balkus tool scores did not vary dramatically across Ayton risk classes (Table 3, Fig. 2) and thus it failed to distinguish between those 3 classes regardless of the cutoff point used.

**Table 3:** Evaluation results of raw Balkus scoring in assessing risk based on the Ayton tool in AGYW (n = 971; 2011–2012).

Cutoff	Sensitivity	Specificity	PPV	NPV
Almost no risk				
$\ge$ 1	0.00 (0.00, 0.01)	1.00 (0.99, 1.00)	0.41 (0.12, 0.83)	0.48 (0.48, 0.48)
$\ge$ 2	0.00 (0.00, 0.01)	1.00 (0.99, 1.00)	0.41 (0.12, 0.83)	0.48 (0.48, 0.48)
$\ge$ 3	0.57 (0.52, 0.62)	0.21 (0.17, 0.24)	0.44 (0.42, 0.46)	0.31 (0.27, 0.35)
$\ge$ 4	0.85 (0.81, 0.88)	0.05 (0.03, 0.07)	0.49 (0.48, 0.50)	0.23 (0.16, 0.32)
$\ge$ 5	0.94 (0.92, 0.96)	0.00 (0.00, 0.01)	0.51 (0.50, 0.51)	0.04 (0.01, 0.19)
$\ge$ 6	1.00 (0.99, 1.00)	0.00 (0.00, 0.01)	0.52 (0.52, 0.52)	0.32 (0.08, 0.77)
Low risk				
$\ge$ 1	0.00 (0.00, 0.01)	1.00 (0.99, 1.00)	0.39 (0.08, 0.78)	0.62 (0.62, 0.62)
$\ge$ 2	0.00 (0.00, 0.01)	1.00 (0.99, 1.00)	0.39 (0.08, 0.78)	0.62 (0.62, 0.62)
$\ge$ 3	0.69 (0.65, 0.73)	0.32 (0.27, 0.37)	0.38 (0.36, 0.41)	0.63 (0.58, 0.67)
$\ge$ 4	0.92 (0.88, 0.93)	0.11 (0.08, 0.15)	0.39 (0.38, 0.40)	0.67 (0.58, 0.75)
$\ge$ 5	0.98 (0.96, 0.99)	0.03 (0.02, 0.05)	0.38 (0.38, 0.39)	0.66 (0.48, 0.81)
$\ge$ 6	1.00 (0.99, 1.00)	0.00 (0.00, 0.01)	0.38 (0.38, 0.38)	0.62 (0.22, 0.92)
High risk				
$\ge$ 1	0.00 (0.00, 0.01)	1.00 (0.96, 1.00)	0.02 (0.00, 0.10)	0.90 (0.90, 0.90)
$\ge$ 2	0.00 (0.00, 0.01)	1.00 (0.96, 1.00)	0.02 (0.00, 0.10)	0.90 (0.90, 0.90)
$\ge$ 3	0.75 (0.72, 0.78)	0.87 (0.78, 0.93)	0.38 (0.27, 0.51)	0.97 (0.96, 0.97)
$\ge$ 4	0.92 (0.90, 0.94)	0.32 (0.22, 0.42)	0.13 (0.11, 0.14)	0.97 (0.96, 0.98)
$\ge$ 5	0.99 (0.98, 0.99)	0.16 (0.09, 0.25)	0.11 (0.11, 0.12)	0.99 (0.98, 1.00)
$\ge$ 6	1.00 (1.00, 1.00)	0.01 (0.00, 0.06)	0.10 (0.10, 0.10)	0.99 (0.95, 1.00)

As the Balkus score cutoff value increased, in all three AGYW risk classes, sensitivity and PPV estimates increased, while specificity and NPV estimates that decreased (Table 3, Fig. 2). Based on sensitivity, specificity, PPV, and NPV, the value of 3 was the best cutoff for the raw Balkus scores in all three Ayton risk classes, suggesting that the Balkus tool score does not distinguish well between classes of AGYW risk identified in the Ayton tool.

**Figure 3:**
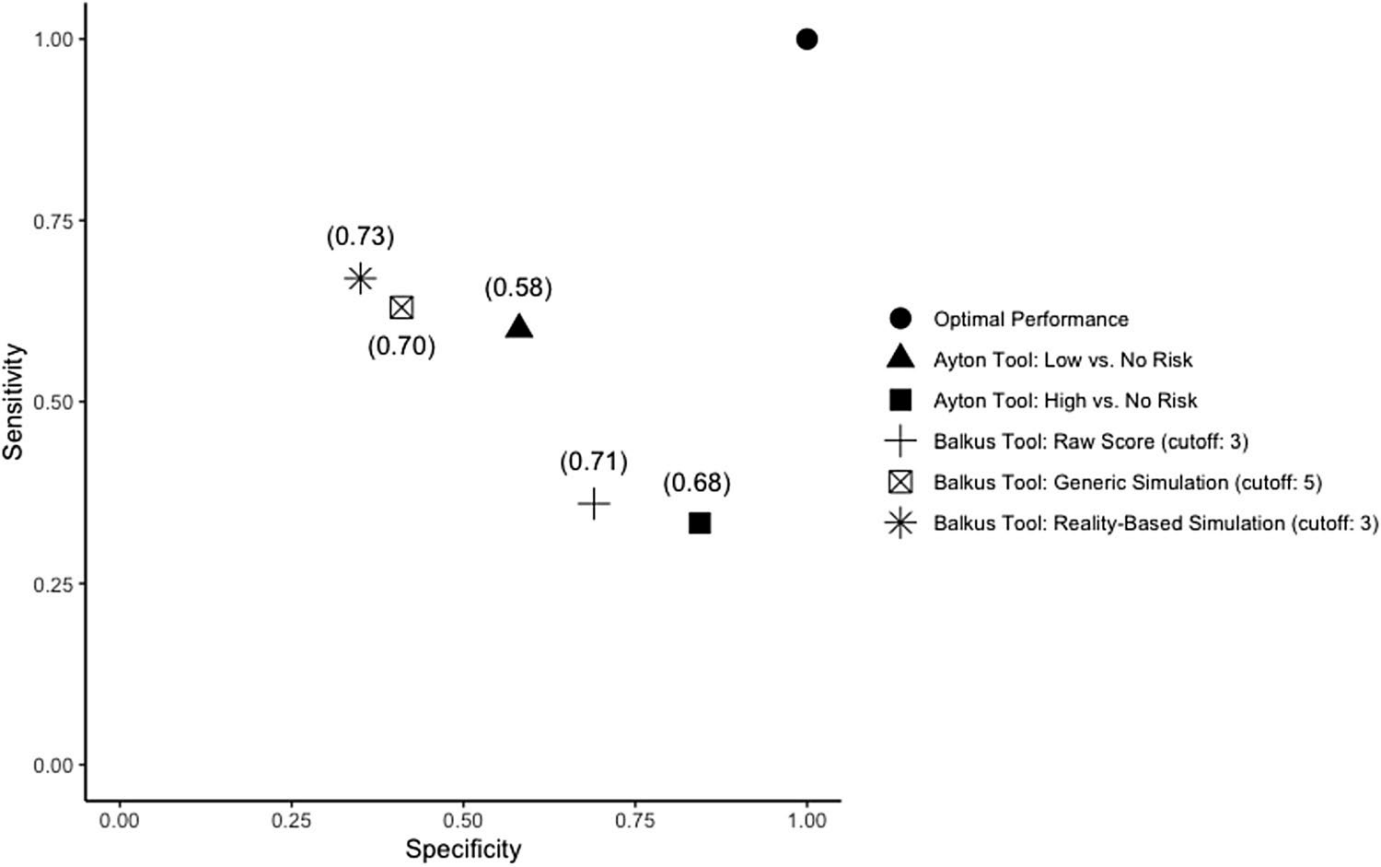
Performance of Ayton tool risk classes as well as all Balkus evaluations compared with optimal performance, defined by 100% sensitivity and 100% specificity. Distances to the optimal performance point are displayed in parentheses.

The comparison of the Balkus and Ayton tools, across all Balkus evaluations and Ayton classes, was evaluated by calculating the distance to the point of optimal performance, the distance from 100% sensitivity and 100% specificity in predicting HIV acquisition (Fig. 3). This comparison revealed the best performance in predicting 1-year HIV acquisition in the Ayton low risk class (sensitivity: 0.60, 95%CI: 0.32–0.84; specificity: 0.58, 95%CI: 0.55–0.61), and slightly better performance in the Ayton high risk class (sensitivity: 0.33, 95%CI: 0.07–0.70; specificity: 0.84, 95%CI: 0.81–0.87) compared with the all Balkus tool evaluations (Supplementary Table S2 and Table 2, Fig. 3). Overall, as can be seen in Fig. 3, the Ayton tool performance in terms of sensitivity and specificity is the closest to the optional performance point (1,1).

## Discussion

AGYW are an underserved and high-risk population in the HIV epidemic in eastern and southern Africa^12–18^. Vulnerability to HIV acquisition is driven, in part, by power imbalance in relationships with older male partners^19,21,22^ and may be prevented by the administration of PrEP^23–26^. In ongoing efforts to combat the transmission of HIV among AGYW, there is a distinct need for risk assessment tools that capture heterogeneities in AGYW HIV acquisition risk, in addition to the efficient identification of those at high risk of infection^7,15,20,22,25,26^. Risk assessment tools have successfully predicted HIV acquisition among adult African women^4,5^ and the development of such prognostic tools for the AGYW population^27^ may prove effective in breaking the HIV transmission cycle in South Africa, by directing the administration of PrEP to prevent HIV infection among high-risk AGYW.

This is the first study to evaluate the use of the Balkus tool in South African AGYW and to evaluate the Ayton tool, the first risk predictive tool to be developed specifically for the AGYW population. We applied the Balkus risk assessment tool in South African AGYW, identified its deficiencies therein, and adjusted for these insufficiencies with advanced statistical simulation techniques. We further assessed the Ayton tool’s predictive ability in the same cohort, as well as assessed the ability of the Balkus tool to distinguish between Ayton risk classes. Finally, we compared the performance of the Balkus and Ayton tools in AGYW. The Ayton tool consistently outperformed the Balkus tool (in simulated and un-simulated evaluations) in predicting 1-year HIV seroconversions and showed that the Balkus tool was unable to distinguish between AGYW risk classes identified with the Ayton tool.

The Balkus score, when applied to AGYW, misidentified high-risk individuals more often than when the tool was applied among adults, except for generic simulations compared with the HPTN 035 cohort (cutoff of 5). The chances of correct identification of those at high-risk of seroconversion, based on score cutoff designation, was consistently lower for AGYW compared with adults in unstimulated and reality-based simulations, where risk factor prevalence was constrained based on published estimates^20^. All evaluation results indicate that the Balkus tool misidentified low-risk more often among AGYW than among older women. While generic simulations indicated relatively robust tool performance, when we control for the likely prevalence of simulated data (reality-based simulations), or assess the raw Balkus scores, we find lower sensitivity and higher specificity among South African AGYW.

The Ayton tool showed more robust performance than the Balkus tool on all evaluation measures. In the low risk class of AGYW, the Ayton tool was superior to the Balkus tool based on sensitivity and specificity; the Ayton tool also outperformed all Balkus evaluations in the high-risk class of AGYW. The Balkus scores did not distinguish between almost no and low risk classes, and showed only a weak ability to distinguish the high risk class, suggesting AGYW-specific risk characteristics that were incorporated into the development of the Ayton tool are not captured by the Balkus tool. This indicates the Ayton tool’s superior ability to identify AGYW at high risk of infection as well as capture heterogeneities of AGYW risk, which is crucial in the effective implementation of ARV and PrEP interventions.

While the Balkus tool has proven useful in assessing HIV risk in adult women, it requires variables that are typically not applicable to South African AGYW. However, without those variables, the tool is less powerful and established cutoffs limit findings. A high mean sensitivity was observed in generic simulations of the Balkus tool, indicating that in AGYW populations with very high prevalence of marriage-cohabitation, non-monogamy, and STI, the tool may have better performance. However, this effect was significantly diminished in the analysis of raw Balkus scores and reality-based simulations. Overall, these findings provide further support for the use of the Ayton tool as a risk-prediction instrument for South African AGYW, and highlight the shortcomings of the Balkus tool when applied to AGYW.

As our data originate from a randomized controlled trial conducted in schools, we expect some degree of error in our findings due to cluster effects and the small number of seroconversions observed in the data. While randomly simulated variables were used in generic evaluations of the Balkus tool, which could introduce noise, relationships between these variables and other exposures have not been sufficiently studied in AGYW to inform data generation. To address this, raw Balkus scores were evaluated as were reality-based simulations, which used published estimates to guide prevalence estimations in simulations. As discrepancies in tool performance among adult women and AGYW may result from intrinsic age disparities, future work examining HIV risk among AGYW must account for adolescent population characteristics, such as education and family life, which likely vary between populations in prevalence and impact on HIV risk. Further, it is possible that pregnant and HIV infected students are more likely to drop out of school; dynamics influencing school enrollment may have contributed to the low prevalence of HIV in the dataset, which may not fully represent the AGYW population. Notwithstanding these limitations, since most South African AGYW are enrolled in school for at least the first two years of high school, our analysis provides a unique opportunity to understand the AGYW HIV risk trajectory. Future research should focus on validating the Ayton tool to assess its performance in other AGYW populations using predictive analysis, which can assign class memberships in new data. Such advances in prognostic tools enable preventive interventions to determine efficient and optimal treatment administration.

The strength of this research lies in its integration of biological and survey measures obtained from a large prospective cohort in a randomized controlled trial setting and the use of advanced statistical simulation techniques. Prognostic tools provide valuable information for the development of future preventions and the allocation of preventative treatment resources to high-risk populations. The efficacy of such interventions relies on the ability to capture and address heterogeneities in risk. The Ayton tool serves as an improved instrument not only for risk identification, but for understanding risk heterogeneities as well. These results support the use of specialized tools, which consider epidemic severity, age, sex and mode of transmission and can better capture risk heterogeneities. Given heightened vulnerability in southern and eastern Africa and the intergenerational HIV transmission cycle, there is a distinct need for HIV risk assessment tools, such as the Ayton tool, which are designed specifically for AGYW.

## Methods

### Study design and sample

The sample used in our study includes South African AGYW participants, aged 14 to 25 years, who were enrolled in the CAP007 trial in 2010 and were followed up in 2011 and 2012 (n = 1069)^28^. The randomized controlled trial (open-label, matched pair design) tested the effectiveness of a conditional cash incentives (CCIs) program on HIV incidence among students in rural South African High Schools from 2010 to 2012. Informed consent was obtained from all participants prior to enrollment in the study. Students 18 years and older provided consent following a literacy and comprehension assessment, while students < 18 years, provided assent and consent was obtained from the parent or guardian. If the parent or guardian was unavailable, proxy parental consent was obtained from a member of the School Research Support Group (SRSG). Ethical approval was obtained from the University of KwaZulu-Natal Biomedical ethics committee (BF105/010 and Be523/14). All experiments were performed in accordance with relevant guidelines and regulations. From this cohort, we included AGYW CAP007 participants who were HIV-seronegative in 2011, and followed-up in 2012 (n = 1049). Baseline characteristics were obtained from the 2011 data, and 1-year serostatus was identified based on 2012 data. Further details about the trial have been published elsewhere^27–29^.

### The Balkus tool

Balkus et al.^4^ developed an HIV risk-scoring tool from clinical predictive factors among African women aged 18–45 years. Researchers derived the following risk scoring formula: $Risk\;Score = \left( 2 \right)X_{1} + \left( 2 \right)X_{2} + \left( 1 \right)X_{3} + \left( 1 \right)X_{4} + \left( 2 \right)X_{5} + \left( 1 \right)X_{6} + \left( 2 \right)X_{7}$, where ${X}_{1}$ is younger than 25 years, ${X}_{2}$ is not married or living with primary partner, ${X}_{3}$ is alcohol use in the past 3 months, ${X}_{4}$ is no partner provision of financial support, ${X}_{5}$ is primary sex partners have other sex partners (and unknown sex partners), ${X}_{6}$ is presence of curable STI, and ${X}_{7}$ is HSV-2 seropositivity. HIV risk scores ranged from 0 to 11. Further details are published in Balkus et al.^4^.

Raw Balkus scores were computed using age, HSV-2, alcohol use, and financial support variables, derived from data collected in 2011. Alcohol use was operationalized as use in the last year, due to reduced prevalence among AGYW compared to adults. In place of “partner provision of financial or material support”, we approximated financial independence (protective) with sources of spending money among AGYW. This was dichotomized as having or not having spending money.

### Simulations

We applied the Balkus tool to AGYW, identified items that were not applicable to the population and thus not measurable, and used advanced statistical simulations to test the tool’s ability to identify AGYW at high risk of developing HIV. To apply the full Balkus tool, responses to unmeasurable items were randomly and independently simulated (simulated values for different variables did not depend on each other) either via generic binary response data of prevalence varying from 0.0 to 1.0 (‘generic simulations’), or using reality-based responses with prevalence varying between 0.0 and 0.4 (‘reality-based simulation’)^30^. Each type of simulation was repeated 100,000 times, allowing prevalence of simulated variables to independently vary in each replication. Since various risk score cutoff values (for binarization of HIV acquisition risk) were employed in the development and validation of the Balkus tool^4^, the obtained risk scores were binarized and evaluated at all possible scores between 1 and 11.

### The Ayton tool

The Ayton AGYW tool, the first HIV risk scoring tool developed for South African AGYW was built with this cohort of CAP007 participants^27^. Through the use of advanced statistical methods, the tool was able to capture socioeconomic and sexual behavioral dimensions of HIV risk and classify participants into three groups of varying risk.

Using the Ayton tool, developed in Ayton et al.^27^, we classified our sample of AGYW in to 3 risk classes based on data obtained in 2011: those at almost no risk, low risk, and high risk. To assess the Ayton tool’s classification of AGYW based on 2011 data, we compared Ayton tool classification results with data on 1-year HIV serostatus (2012) for each risk class.

### Statistical analysis

The Balkus tool risk scores based on 2011 data were compared with 1-year HIV serostatus (obtained from 2012 data). The same was done for Ayton tool. Sensitivity and specificity were computed along with their respective 95% confidence intervals for every possible score cutoff (Balkus tool) and within each risk class (Ayton tool) and compared. To compute 95% confidence intervals for the sensitivity and specificity, the exact, conservative Clopper Pearson method was used^31^. Using the estimate of HIV prevalence for South African AGYW derived from a population-based nationally representative survey conducted in 2012, positive predictive values (PPV) and negative predictive values (NPV) were also computed. The asymptotic standard logit intervals^32^ were used to compute intervals for the predictive values, and, where appropriate, the adjusted logit intervals^32^ were returned instead to compute intervals for the predictive values.

All analyses were performed in R version 3.4.1.

## Supplementary information

Supplementary information.
