# Supplementary material for: Identification of adolescent girls and young women for targeted HIV prevention: a new risk scoring tool in KwaZulu Natal, South Africa
Source: Sci Rep. 2020 Aug 3;10:13017. doi: 10.1038/s41598-020-69842-x (PMC7400571; doi:10.1038/s41598-020-69842-x)
Supplement: Supplementary file 1 — Supplementary information. [file 41598_2020_69842_MOESM1_ESM.docx]

**Identification of Adolescent Girls and Young Women for targeted HIV prevention:**

**A new risk scoring tool in KwaZulu Natal, South Africa**

Sarah Gabrielle Ayton^1,2,§^, Martina Pavlicova^1^, Quarraisha Abdool Karim^1,3^

^§^ Corresponding author: sarah.ayton@columbia.edu

1 Mailman School of Public Health, Columbia University, New York, United States of America

2 Escuela de Medicina y Ciencias de la Salud, Tecnologico de Monterrey, Monterrey, Mexico

3 Centre for the AIDS Programme of Research in South Africa, Durban, South Africa

**Supplementary Information**

Contents:

Supplementary Table S1

Supplementary Table S2

Supplementary Table S1. Distribution of AGYW and HIV seropositive (at one year) AGYW by Balkus score (n = 971; 2011-2012)

| Score |  | Raw Balkus Score | | |  |
| --- | --- | --- | --- | --- | --- |
|  |  | HIV cases | AGYW | HIV prevalence  (95% CI) | |
| 0 |  | 0 | 1 | 0.00 -- | |
| 1 |  | 0 | 0 | 0.00 -- | |
| 2 |  | 9 | 668 | 0.01 (0.01, 0.03) | |
| 3 |  | 2 | 206 | 0.01 (0.00, 0.04) | |
| 4 |  | 1 | 70 | 0.01 (0.00, 0.09) | |
| 5 |  | 2 | 25 | 0.08 (0.01, 0.28) | |
| 6 |  | 0 | 1 | 0.00 -- | |
| Total |  | 14 | 971 | 0.01 (0.01, 0.02) | |
|  |  |  |  |  | |
| Score |  | Balkus Score Based on 100,000 Generic Simulation | | |  |
|  |  | HIV cases  Mean (95% CI) | AGYW  Mean (95% CI) | HIV prevalence  (95% CI) | |
|  |  |  |  |  |  |
| 0 |  | 0.00 (0.00, 0.00) | 0.13 (0.12, 0.13) | 0.00 (0.00, 0.99) | |
| 1 |  | 0.00 (0.00, 0.00) | 0.12 (0.12, 0.13) | 0.00 (0.00, 0.99) | |
| 2 |  | 1.12 (1.11, 1.13) | 83.76 (83.17, 84.35) | 0.01 (0.00, 0.08) | |
| 3 |  | 1.37 (1.36, 1.38) | 109.51 (108.87, 110.15) | 0.01 (0.00, 0.06) | |
| 4 |  | 2.63 (2.62, 2.64) | 201.61 (200.95, 202.27) | 0.01 (0.00, 0.04) | |
| 5 |  | 3.13 (3.12, 3.14) | 230.51 (229.88, 231.13) | 0.01 (0.00, 0.04) | |
| 6 |  | 2.12 (2.11, 2.13) | 155.74 (155.22, 156.26) | 0.01 (0.00, 0.05) | |
| 7 |  | 2.12 (2.11, 2.13) | 133.12 (132.49, 133.76) | 0.02 (0.00, 0.06) | |
| 8 |  | 0.87 (0.87, 0.88) | 41.00 (40.80, 41.20) | 0.02 (0.00, 0.14) | |
| 9 |  | 0.38 (0.37, 0.38) | 12.13 (12.06, 12.20) | 0.03 (0.00, 0.34) | |
| 10 |  | 0.25 (0.25, 0.25) | 3.24 (3.22, 3.27) | 0.08 (0.00, 0.72) | |
| 11 |  | 0.00 (0.00, 0.00) | 0.13 (0.12, 0.13) | 0.00 (0.00, 0.99) | |
| Total |  | 14 | 971 | 0.01 (0.01, 0.02) | |
|  |  |  |  |  | |
| Score |  | Balkus Score Based on 100,000 Reality-Based Simulation | | |  |
|  |  | HIV cases  Mean (95% CI) | AGYW  Mean (95% CI) | HIV prevalence  (95% CI) | |
|  |  |  |  |  |  |
| 0 |  | 0.00 (0.00, 0.00) | 0.51 (0.51, 0.51) | 0.00 (0.00, 0.97) | |
| 1 |  | 0.00 (0.00, 0.00) | 0.13 (0.13, 0.13) | 0.00 (0.00, 0.99) | |
| 2 |  | 4.61 (4.60, 4.62) | 342.29 (341.78, 342.81) | 0.01 (0.00, 0.03) | |
| 3 |  | 2.18 (2.17, 2.18) | 191.02 (190.70, 191.33) | 0.01 (0.00, 0.04) | |
| 4 |  | 3.07 (3.06, 3.08) | 233.24 (232.96, 233.52) | 0.01 (0.00, 0.04) | |
| 5 |  | 2.24 (2.24, 2.25) | 117.26 (117.05, 117.46) | 0.02 (0.00, 0.07) | |
| 6 |  | 0.93 (0.93, 0.94) | 56.20 (56.03, 56.36) | 0.02 (0.00, 0.11) | |
| 7 |  | 0.71 (0.71, 0.72) | 22.95 (22.86, 23.03) | 0.03 (0.00, 0.22) | |
| 8 |  | 0.17 (0.17, 0.18) | 5.75 (5.72, 5.77) | 0.03 (0.00, 0.53) | |
| 9 |  | 0.07 (0.07, 0.07) | 1.42 (1.41, 1.43) | 0.05 (0.00, 0.90) | |
| 10 |  | 0.02 (0.02, 0.02) | 0.23 (0.23, 0.23) | 0.07 (0.00, 0.99) | |
| 11 |  | 0.00 (0.00, 0.00) | 0.01 (0.01, 0.01) | 0.00 (0.00, 1.00) | |
| Total |  | 14 | 971 | 0.01 (0.01, 0.02) | |

**Supplementary Table S2. Evaluation results of Balkus scoring in predicting HIV serostatus at one year in AGYW (n = 971; 2011-2012)**

| Cutoff |  | Raw Balkus Score | | | |
| --- | --- | --- | --- | --- | --- |
|  |  | Sensitivity | Specificity | PPV* | NPV* |
| $\geq$ 1 |  | 1.00 (0.77, 1.00) | 0.00 (0.00, 0.01) | 0.11 (0.09, 0.12) | 0.19 (0.04, 0.55) |
| $\geq$ 2 |  | 1.00 (0.77, 1.00) | 0.00 (0.00, 0.01) | 0.11 (0.09, 0.12) | 0.19 (0.04, 0.55) |
| $\geq$ 3 |  | 0.36 (0.13, 0.65) | 0.69 (0.66, 0.72) | 0.13 (0.07, 0.24) | 0.89 (0.85, 0.92) |
| $\geq$ 4 |  | 0.21 (0.05, 0.51) | 0.90 (0.88, 0.92) | 0.23 (0.10, 0.45) | 0.90 (0.87, 0.92) |
| $\geq$ 5 |  | 0.14 (0.02, 0.43) | 0.97 (0.96, 0.98) | 0.43 (0.16, 0.74) | 0.90 (0.87, 0.91) |
| $\geq$ 6 |  | 0.00 (0.00, 0.23) | 1.00 (0.99, 1.00) | 0.81 (0.45, 0.96) | 0.89 (0.88, 0.91) |
|  |  |  |  |  |  |
| Cutoff |  | Generic-simulated Balkus Score | | | |
|  |  | Sensitivity | Specificity | PPV* | NPV* |
| $\geq$ 1 |  | 1.00 (0.77, 1.00) | 0.00 (0.00, 0.00) | 0.11 (0.09, 0.12) | 0.12 (0.02, 0.50) |
| $\geq$ 2 |  | 1.00 (0.77, 1.00) | 0.00 (0.00, 0.00) | 0.11 (0.09, 0.12) | 0.13 (0.02, 0.51) |
| $\geq$ 3 |  | 0.92 (0.67, 0.98) | 0.09 (0.07, 0.10) | 0.11 (0.09, 0.13) | 0.62 (0.39, 0.81) |
| $\geq$ 4 |  | 0.82 (0.56, 0.94) | 0.20 (0.18, 0.22) | 0.12 (0.09, 0.15) | 0.74 (0.56, 0.87) |
| $\geq$ 5 |  | 0.63 (0.37, 0.83) | 0.41 (0.38, 0.43) | 0.13 (0.09, 0.19) | 0.87 (0.75, 0.93) |
| $\geq$ 6 |  | 0.41 (0.19, 0.67) | 0.65 (0.62, 0.67) | 0.15 (0.08, 0.28) | 0.89 (0.83, 0.92) |
| $\geq$ 7 |  | 0.26 (0.09, 0.53) | 0.81 (0.78, 0.83) | 0.21 (0.09, 0.41) | 0.89 (0.86, 0.92) |
| $\geq$ 8 |  | 0.11 (0.02, 0.37) | 0.94 (0.93, 0.95) | 0.34 (0.13, 0.63) | 0.89 (0.87, 0.91) |
| $\geq$ 9 |  | 0.04 (0.00, 0.29) | 0.98 (0.98, 0.99) | 0.54 (0.25, 0.83) | 0.89 (0.88, 0.91) |
| $\geq$ 10 |  | 0.02 (0.00, 0.26) | 1.00 (0.99, 1.00) | 0.75 (0.39, 0.94) | 0.89 (0.88, 0.91) |
| $\geq$ 11 |  | 0.00 (0.00, 0.23) | 1.00 (1.00, 1.00) | 0.88 (0.50, 0.98) | 0.89 (0.88, 0.91) |
|  |  |  |  |  |  |
| Cutoff |  | Reality-based simulated Balkus Score | | | |
|  |  | Sensitivity | Specificity | PPV* | NPV* |
| $\geq$ 1 |  | 1.00 (0.77, 1.00) | 0.00 (0.00, 0.00) | 0.11 (0.09, 0.12) | 0.15 (0.03, 0.52) |
| $\geq$ 2 |  | 1.00 (0.77, 1.00) | 0.00 (0.00, 0.01) | 0.11 (0.09, 0.12) | 0.16 (0.03, 0.53) |
| $\geq$ 3 |  | 0.67 (0.39, 0.88) | 0.35 (0.32, 0.38) | 0.12 (0.09, 0.17) | 0.89 (0.79, 0.94) |
| $\geq$ 4 |  | 0.52 (0.25, 0.77) | 0.55 (0.52, 0.58) | 0.13 (0.09, 0.21) | 0.90 (0.83, 0.94) |
| $\geq$ 5 |  | 0.30 (0.10, 0.58) | 0.79 (0.77, 0.82) | 0.17 (0.08, 0.32) | 0.89 (0.86, 0.92) |
| $\geq$ 6 |  | 0.14 (0.03, 0.41) | 0.91 (0.89, 0.93) | 0.21 (0.08, 0.49) | 0.90 (0.87, 0.91) |
| $\geq$ 7 |  | 0.07 (0.01, 0.33) | 0.97 (0.96, 0.98) | 0.36 (0.14, 0.71) | 0.89 (0.87, 0.91) |
| $\geq$ 8 |  | 0.02 (0.00, 0.26) | 0.99 (0.99, 1.00) | 0.62 (0.29, 0.89) | 0.89 (0.88, 0.91) |
| $\geq$ 9 |  | 0.01 (0.00, 0.24) | 1.00 (0.99, 1.00) | 0.80 (0.43, 0.96) | 0.89 (0.88, 0.91) |
| $\geq$ 10 |  | 0.00 (0.00, 0.23) | 1.00 (1.00, 1.00) | 0.87 (0.49, 0.98) | 0.89 (0.88, 0.91) |
| $\geq$ 11 |  | 0.00 (0.00, 0.23) | 1.00 (1.00, 1.00) | 0.88 (0.50, 0.98) | 0.89 (0.88, 0.91) |
| *Computed with 11.4% prevalence estimate of HIV in South African AGYW (Mabaso et al., 2018) | | | | | |
